# Supplementary material for: Impaired respiration elicits SrrAB-dependent programmed cell lysis and biofilm formation in Staphylococcus aureus
Source: eLife. 2017 Feb 21;6:e23845. doi: 10.7554/eLife.23845 (PMC5380435; doi:10.7554/eLife.23845)
Supplement: Supplementary file 1. — DOI: http://dx.doi.org/10.7554/eLife.23845.019 [file elife-23845-supp1.docx]

| Supplementary File 1: Oligonucleotides used in this study  **RT- PCR primers** | |
| --- | --- |
| *tarO* For | AATTGCCGCTGCCTTAGTAGTT |
| *tarO* Rev | TGTACCCATTGGCAACGAAA |
| *tarA* For | GATGGGACAGGAGTAGTCAAAGCT |
| *tarA* Rev | ACCAGGTATACGATGCGCTAGAG |
| *tarH* For | GGCTTGTTGGCATCAATGG |
| *tarH* Rev | CCGCCAATGATATTGCTCAA |
| *tarB* For | ATGTGTCTGACAAGGCAATGGT |
| *tarB* Rev | CACTAAGTAAAAATCCGTCGCTTGA |
| *atlA* For | GGTGCAGTCGGTAACCCTAGAT |
| *altA* Rev | TGAACGTGCAAATGAAGCATAGT |
|  |  |
| **Cloning primers** |  |
|  |  |
| pLL39_yeast F | GCCCAATCACTAGTGAATTCCCGAAGCTTAGTTACGCTAGGGATAACAG |
| yeast_srrPro R | atatcttggatgtgtcattaaCTATATTACCCTGTTATCCC |
| yeast_srrPro F | GGGATAACAGGGTAATATAGttaatgacacatccaagatat |
| srrAB_pLL39 R | GGTAATAAAAAAGCTTGCATGCCTGCAGGttttattctggttttggtag |
|  |  |
